# Rationalizing Science: A Comparative Study of Public, Industry, and Nonprofit Research Funders

**DOI:** 10.1007/s11024-018-9352-6

**Published:** 2018-05-02

**Authors:** Noomi Weinryb, Maria Blomgren, Linda Wedlin

**Affiliations:** 1 Academy of Public Administration, Södertörn University, 141 89 Huddinge, Sweden; 2 Department of Business Studies, Uppsala University, Box 513, 751 20 Uppsala, Sweden

**Keywords:** Research funding, Organizational institutionalism, World society, Nonprofit research funders, Commercialization

## Abstract

In the context of more and more project-based research funding, commercialization and economic growth have increasingly become rationalized concepts that are used to demonstrate the centrality of science for societal development and prosperity. Following the world society tradition of organizational institutionalism, this paper probes the potential limits of the spread of such rationalized concepts among different types of research funders. Our comparative approach is particularly designed to study the role and position of nonprofit research funders (NPF), a comparison that is relevant as NPF could potentially be shielded from such rationalized pressures given their lack of profit gaining motives. By making a qualitative interview-based investigation we are able to describe how research funders rationalize their contributions to society at large, as well as their obligations to the researchers they fund. Four types of research funders are compared—independently wealthy philanthropists, fundraising dependent nonprofits, public agencies, and industry. We find that NPF, and especially philanthropists, are the least commercially geared type of funder, but that philanthropists also express least obligations to researchers funded. This is in sharp contrast to public research funders who, even more than industry, employ commercially geared rationalizations. We also find that both public and corporate funders express obligations to the researchers they fund. Our results indicate that there are limits to the spread of commercially tinted rationalizations among NPF, but that this does not necessarily mean an increased sense of obligations to the researchers funded, and by extension to the integrity of scientific pursuit.

## Introduction

As contemporary funding of science has become ever more project-based, private sources of funds have gained importance. This expansion of private funders includes both industry and nonprofit research funders (NPF) (Gulbrandsen and Smeby 2005; Stephan 2012). The increasing relevance of alternative funding sources stems to a large extent from the changing principles of public science spending and the growing adherence to “market-based” solutions for resource allocation. The idea that all types of activities should be organized through the invisible hand of the market has altered public policy across the globe (Hood 1991), including public spending on science and research. The after-war period, and perhaps even more notably from the 1980s and onwards, has been characterized by what Geuna (2001: 617) calls a “contractual-oriented” approach to research funding whereby national economic development and competitiveness is promoted as the primary aim of universities, and competitive mechanisms for resource allocation have become a defining feature of national science systems (see also Whitley and Gläser 2007; Whitley et al. 2010; Auranen and Nieminen 2010). This development has opened up a space for private actors and interests to become significant actors in science funding, including NPF.

As a part of this development, commercialization and economic growth have increasingly become important concepts as means to argue for, and invest resources in, science and research. The spread of these concepts mirrors the development of a research policy debate, across Europe and elsewhere, which places science as a key component in the promotion of economic, social, and cultural growth and prosperity for the general benefit of society (Wedlin and Nedeva 2015; Buckner 2017). In this research policy debate, direct efforts to encourage and strengthen the links between universities and other actors, such as industry, and to increase the “usefulness” of science has been developed, not least through policies aimed to “commercialize” science and research findings. Although the overarching rationale for these commercialization processes may be similar, the manner in which such processes take place vary between different places (Slaughter and Cantwell 2012). Commercialization efforts include patenting important scientific results, creating spin-offs or new businesses, or in other ways contributing to innovation policies and practice (Engwall and Weaire 2008). The content of these interactions are assumed to play a crucial role for strengthening the innovation capacity of contemporary society, and are focused on increasing interaction between the university and other actors surrounding it, primarily commercial (Etzkowitz and Leydesdorff 2000; Nowotny et al. 2001).

Given this development, commercial benefits and economic growth can be considered predominant rationalized concepts of science and scientific research, entering into a multitude of science organizations in various ways (Drori et al. 2006; Drori et al. 2003; Schofer and Meyer 2005), not the least among funders of science. Braun (1998) notes that the structures, norms and interests articulated by funders – i.e., the rationalizations of research funders – have an influence on their funding decisions and, in the longer run, also on scientific output. Rationalizations of science are thus assumed to be relevant for the cognitive shaping of science at large, particularly as they enter into the funding and funding rationales of research funders.

While previous research, albeit sometimes taking different normative positions, indicates that both public and private funders express rationalizations of commercialization and economic growth (Glenna et al. 2011; Goldfarb 2008; Sismondo 2007; Etzkowitz and Leydesdorff 2000; Nowotny et al. 2001), we know little about the rationalizations of nonprofit research funders (NPF). Although NPF have become increasingly important in many national science systems in recent years (Stephan 2012; Azoulay et al. 2011; Geuna 2001), their role and position in the contemporary funding landscape has not been systematically explored. Given their lack of profit-gaining motives (Weisbrod 1991; Hansmann 1980), are they perhaps shielded from the market-based pressures to rationalize scientific output in commercial terms? If so, are NPF using alternative ways to motivate and rationalize their research funding practices, and do they relate differently than other types of funders to the researchers they fund? The aim of this paper is to investigate the rationalizations of different types of research funders in order to explore the spread and limits – if any – of the commercialization-focused rationalizations of science among research funders. For this purpose, we focus particularly on NPF in comparison to public and industry funders. Our specific research questions therefore read: How do NPF, public, and industry research funders rationalize their contributions to society, and how do they rationalize their obligations to the researchers they fund?

## Conceptual Framework

The point of departure of this paper is organizational institutionalism, and especially its world society stream, which stipulates that society is increasingly rationalized and organized (Meyer et al. 1997; Drori 2008). Organizations become more similar to each other with time, as demands to be legitimate entail institutional pressures to adhere to specific ideas and norms about what is appropriate (Meyer and Rowan 1977). The development of world society entails a tendency of all sorts of organizations to become more focused on commercialization and managerialism, adopting similar structures and goals, despite often operating in entirely different fields. It is as if the all-encompassing idea of what it means to be “an organization” becomes generalizable to all types of organizations (Bromley and Meyer 2014; Meyer and Bromley 2013). The world society literature has been particularly focused on science, the spread of science, and its role as both being rationalized and rationalizing others (Meyer et al. 1997; Drori and Meyer 2006; Drori et al. 2003). The spread of the rationalized concepts of commercialization and economic growth in science may be viewed as attempts to rationalize funding practices and gain legitimacy, regardless of organizational form or actual resource provision. A substantial body of empirical work in organizational institutionalism has pointed to the increased homogeneity in mass education, scientific pursuit, university rankings, and recruitment of international PhD students (Drori et al. 2003; Schofer and Meyer 2005; Taylor and Cantwell 2015; Wedlin 2007; Meyer et al. 1992). This body of work can be used to reinforce the idea that within the field of science, different types of scientific actors, pursuing different scientific traditions, in different locations and with different scope, all become similar in their rationalization of their activities.

Differing from studies chronicling the spread of isomorphism in science and science organizations, most available studies of NPF assume their distinctiveness. Such is the case both in historical studies of philanthropic foundations and their funding of science, and in accounts of, for example, the development of specific disease-related nonprofit patient groups. This reasoning stems from ideas that nonprofits provide a form of organizing that bridges an efficiency gap, which public agencies and corporations are unable to cover, and that precisely this property makes them essentially different from those other types of organizations (Smith and Gronbjerg 2006; Coase 1937; Alchian and Demsetz 1972). From this perspective, the existence of NPF can be explained by the dissatisfaction with political decisions of the non-median voter, who takes the production of public goods (such as research funding) into her or his own hands (Weisbrod 1991). Another theoretically related explanation focuses on contractual difficulties in providing a certain good on the private for-profit market (such as some types of research funding) as resulting in the need to create nonprofits (Hansmann 1980). Based on such explanations, it is the “nonprofitness” that will make NPF different in their rationalization of their funding practices, despite the norms and rationales that impregnate the funding environment in which they operate. This literature builds on contingency ideas tied to “nonprofitness” that may have consequences for how funders relate to their grantees. For example, previous research has pointed to how some NPF have used their freedom to create a long-term engagement with the researchers funded, and have in this manner managed to fund more high-impact research in comparison to public funders (Azoulay et al. 2011).

Studies of philanthropists often point out the unique contributions of old and famous research foundations such as Carnegie, Rockefeller, Russel Sage, and Ford (Hammack 2000; Hall 2006; Lagemann 1999; Fleishman 2009). There are also papers analyzing philanthropists’ special influence on the development of specific branches of science, for example, the Rockefeller Foundation and its role in shaping such diverse academic disciplines as economics, physiology, social science, physical sciences, and medicine (Bulmer and Bulmer 1981; Abir-Am 1982; Craver 1986; Cueto 1990; Fisher 1983; Weindling 1997). Other studies describe the history of specific research foundations shaping science in other parts of the world (Benner and Sörlin 2007) and the contributions of philanthropic funding to higher education (Thelin and Trollinger 2014). Moving from funding organization to funding cause, studies of patient groups often focus on one type of disease at a time, and investigate the importance of patient activism in the context of research funding (Rabeharisoa and Callon 2002; Epstein 1995; Panofsky 2011).

In sum, all of these NPF case studies indicate that the specific resource provision of NPF, being “nonprofit,” will make these funders bring about distinctive contributions to the research funding landscape. However, following the tenets set out by organizational institutionalism, and particularly its world society tradition, there are reasons to argue that NPF would be equally adherent to predominant rationalized ideals as any other type of funder (Meyer et al. 1997; Bromley and Meyer 2014). Several studies of nonprofits, although not focused on research funding, indicate that they become increasingly similar to the industry-like ideals of what it means to be “an organization” in order to gain legitimacy rather than necessarily to pursue actual profits (Hwang and Powell 2009; Maier et al. 2016). By comparing NPF to other public and industry research funders, we are able to explore the potential limits of the spread of rationalized concepts such as commercialization and economic growth among funders of science. This enables us to see if there is a functional distinction that may make NPF different from other funders, or if they are equally rationalized.

## Research Design

To explore the spread and possible limits of world society, and the potential distinctiveness of NPF in relation to other funders, we focus our attention on comparing how the funders themselves argue and rationalize their role in society and their relationship to science and researchers. In this we are building on the work of Braun (1998), which indicates that the rationalizations employed by funders will be relevant for their funding practices. We therefore mean that the way funders choose to formulate their rationalizations, or bluntly speaking - the way they talk about their activities and their own role - is indicative of their broader general stances towards the scientific pursuit and of their understanding of their own role in the funding landscape.

To investigate these rationalizations, we created descriptive qualitative accounts based on funders’ explanations of what they do and what role they take in funding science, thus explicating their funding rationales. We operationalize these rationales in two ways. Firstly, we ask: How do funders rationalize their contributions to society, and how do they more specifically relate to notions of commercialization of research and the relevance of science for economic growth? This is intended to be an indication of the extent to which the funding rationales of contemporary world society are used by the funders, and thus demonstrating the general adherence to the competitive market-based and commercially focused rationalizations of science. We here refer both to commercialization as profit-gaining on an organizational level, as well as economic growth in society at large.

Secondly, to supplement their specific rationalization of commercialization and economic growth, we also asked the funders to elaborate on their relations to the scientific field, and more specifically how they understand their obligations to the researchers that they fund. This is a way to understand, indirectly, the funders’ perceptions of science and their adherence to scientific ideals and principles. While not in direct opposition to commercialization and economic rationalities, obligations to researchers can be understood as an alternative way to understand the funders’ rationales and arguments for science and research funding, focusing primarily on general scientific norms and the inherent value of independent scientific pursuit (Merton 1973).

When conducting our empirical study, we have worked with the formulation “accountability to grantees” when referring to obligations to the researchers. We have done this to entice the funders to formulate their relations and obligations to individual scientists and their work, as an indication of their views on the scientific pursuit. This should not be confused with a more common notion of accountability, where the direction would be the opposite: those receiving grants are to be held accountable to the funder. A proliferation of this latter type of accounts has been interpreted as a strong indication of world society, and this trend has been described in-depth by several excellent studies (Musselin 2014; Benner and Sandström 2000; Jang 2005; Geuna and Martin 2003; Hicks 2012). To further our investigation into the spread of world society, we are aware that our study could have benefited from also including this type of empirical material. However, given that we know that the spread of audit-based measures is common, also among nonprofit funders at large (Hwang and Powell 2009; Maier et al. 2016), we find it even more important to provide an additional perspective by asking about funders’ obligations to their grantees. As we are interested in another part of world society - the commercialization of science rather than the audit explosion (Power 1997) - we believe that a focus on the obligations, or accountability, of the funders themselves to their grantees can say something else than a focus on the spread of accounting practices. By contrasting the potential distinctiveness of NPF with the world society tradition, we can compare funders’ potentially commercial rationalizations of their research funding to their rationalizations of their role in the larger science production system. How do these funders talk about the researchers’ influence on their own role? Do they perceive themselves as isolated units capable of free action, or as rationalized nodes on which the larger scientific pursuit is dependent?

As it is the specific manner of resource provision that potentially distinguishes NPF from other types of funders, we followed a comparative scheme which distinguishes industry and public research funders from two ideal types of NPF: independently wealthy philanthropists – both foundations and individuals, and fundraising dependent nonprofit organizations (Weinryb 2015). These two types of NPF have their own distinct ways of obtaining funds, and their income sources are also different from the tax-based public agencies and the sales- and investment-based industry. Together these four types of funders provide a classification based on the ideal type resource provision of the research funders. This typology of research funders is presented in Table 1.

**Table 1:** A resource-based typology of research funders

Funding	Form of ownership	Type of funder
Independent wealth	Private—nonprofit	Philanthropist (individual and foundation)
Funds raised continuously	Private—nonprofit	Fundraising dependent nonprofit
Tax	Public	Public funder
Sales and investments	Private—for-profit	Industry funder

In order to find a context where all four types of research funders (see Table 1) were present and would be accustomed to communicate about their funding practices, our research design was based on a purposeful extreme case selection. We chose to make a cross-sectional comparison in a funding area where we knew NPF had been particularly active, and where all funders were likely to have had to communicate about their funding practices, namely, funding of human embryonic stem cell research (Fossett 2009). Stem cells are cells that can morph into other types of cells, and human embryonic stem cells, which are derived from leftover IVF-embryos, can potentially morph into any other type of cell (Thomson et al. 1998). Human embryonic stem cells have since they were first discovered in 1998 been the target of many hopes from patient communities, researchers, and policy makers alike (Korobkin and Munzer 2007). They have also attracted a lot of negative attention, as their existence is based on the destruction of a potential life, thus activating all the classical anti-abortion arguments of the pro-life movement (Gottweis et al. 2009). The empirical setting of human embryonic stem cell research funding is suitable for studying NPF, as legislative limitations on public research funding have increased the role of NPF in this research area (Weinryb and Bubela 2016). In the United States (US), federal funding was severely restricted under George W. Bush, and also the European Union (EU) has implemented severe restrictions as to the use of federal means for some parts of the research. Instead, public funders on the state (US) and national (EU) level have stepped in and together with industry and NPF made up for federal funding restrictions. This has created a multifaceted funding landscape where many different types of funders interact and co-fund research. But targeted research funding of human embryonic stem cells have not only taken place in the EU and US. Also in East Asia human embryonic stem cell research has received a lot of both positive and negative attention, partially because of a research fraud and funding scandal (Gottweis and Triendl 2006), making it a relevant point of inclusion in any type of research funding study of human embryonic stem cells. In sum, the attention that has been given to this funding area (Scott et al. 2010, 2011; see also Furman et al. 2012), and the inclusion of NPF in it, makes it particularly suitable given the aim of our study.

## Data Collection and Analysis

Given the cross-national character of contemporary scientific research, we chose to create a sample of funders of human embryonic stem cell research spanning across three global centers of stem cell research – the US, the EU, and East Asia. We used PubMed to find the most engaged research funders of human embryonic stem cell research from three societies particularly active in human embryonic stem cell research funding– California, Sweden, and South Korea. In 2011/2012 we searched all the publications of this research that had come out of relevant research institutes located in the three societies and read about, listed, and coded all funders that were thanked in the publications. Based on this list, we manually performed internet searches to assess which funders were explicitly funding this research through some kind of public communication. In addition, a few industry funders were added through consultations with experts in the field, as these funders were not publication-focused. When contacting the sampled funders, we specifically asked about the possibility to interview directors who could represent the funder’s perspective. As an introductory sentence, every interview began with a request that the respondents should reply to the interview questions in the capacity of representing the research funder. Their replies were rationalizations of the work of the funder, and those interpretations were the very phenomenon we wished to investigate.

We interviewed 83 individuals representing 51 direct research funders and we made 18 additional interviews with indirect research funders and biomedical experts in the subject area. These 18 additional interviews were used to get a deepened understanding of the field at large. Indirect research funders were those who had only funded the studied type of research as a component of other research funding, or had done it by directing public agencies that in turn funded the research, but not distributed the funding themselves. In four instances individuals represented two different direct funding organizations at the same time. They were then interviewed separately about each funding organization they represented, and the interviews were counted as separate interviews. Out of all the funders we contacted, 17 potentially relevant funders declined participation in the study. In each society, the expertise of the other participating funders was used to assess the importance of the non-participating funders, and none of them was deemed to be as central to the field as to directly affect our understanding of the rationalizations of the funders participating in the study. In total we made 101 interviews. For a detailed breakdown of respondents representing direct research funders, see Table 2.

**Table 2:** Distribution of direct research funders in sample

Type of funder	Individuals/organizations
Philanthropist	16/12
Fundraising nonprofit	27/17
Public funder	21/11
Industry funder	19/11
Total	83/51

All direct research funders in the sample were interviewed about their contributions to society and obligations to researchers funded. Interviews were done in personal meetings or on the phone, except three that were done via email. The recorded interviews were transcribed verbatim, and each transcript was reviewed in detail, listening to the recording and reading the transcript simultaneously. We made a qualitative coding of the responses, carefully analyzing patterns in the material. The coding was iterative (Saldaña 2012; Eisenhardt 1989), and we moved intermittently between the interview transcripts and the coding scheme. We specifically searched for rationalizations of contributions to society related to commercialization or the lack of those, also in the form of economic growth. In terms of obligations to researchers, we looked for statements indicating the nature of funder obligations to grantees, or lack thereof. The coding was done in three rounds. In the first round, we simply gathered all relevant statements on the investigated rationalizations, categorized per funder. As a second step, we looked for patterns in the statements, discerning structures in the rationalizations that might correspond to our resource-based classifications system of funders. In the final step, we returned to our conceptual framework, carefully analyzing how our findings could enrich the current knowledge on the rationalizations employed by different types of research funders, comparing NPF to public and industry funders.

## Findings

To facilitate the ease of access to our qualitative interview data, we have chosen to organize the presentation of our results as follows. We will introduce each type of funder in a separate subsection, employing our four-type funder typology: independently wealthy philanthropists (NPF), fundraising dependent nonprofits (NPF), public funders, and industry funders. In each subsection, we will begin with representative quotes on contributions to society, which will then be followed by representative quotes on obligations to researchers. The quotes are selected to provide a sense of both the richness of the interviews and the patterns we have identified. To let the data speak as much as possible, we will accompany each subsection with sparse but carefully selected comments, and we will end each subsection with a brief summary of the patterns identified. The results will then be discussed and compared more in depth in the discussion section of the paper.

### NPF: Independently Wealthy Philanthropists

**Contributions to society:** Our interviews indicate that philanthropists seemed to care very little about commercial applications of the research they funded. Instead, they were articulated in their promotion of basic research:

“we contribute of course at a very basic level, so it is difficult to go out on the town and be proud by seeing something [that we have achieved], instead we are satisfied with, so far in any case, supporting basic research and believing that this positively affects the wellbeing of humanity in the long run. We are not waiting for any breakthroughs, or some Nobel laureates or the like, we have no such hopes, but [what we want] it is to support basic research as well as possible.” Interviewee 68, Philanthropist

Philanthropists emphasized the value of creative research for its own sake:

“It appeared to us that the most significant research in the country was going on in California. Not just in stem cell biology, but in terms of creative research. So we were - we became one of the largest funders of research in California because we - not because we made a conscious decision to do that, but because that’s where the most creative research is going on.” Interviewee 21, Philanthropist

But in the very long run, this basic research may improve society:

“I believe that prosperity is built through research and innovation, but above all by educating competent people and we are one of the key players who contribute to that. But we are not late in the innovation phase, but we are very early. Our whole ambition is to build basic skills both in research and in education to build a better society.” Interviewee 60, Philanthropist

However, it was important for the philanthropists to emphasize that this improvement of society was very far from a desire to focus on applied research:

“We would never say to a scientist, you must develop a cure.” Interviewee 60, Philanthropist

**Obligations to researchers:** Even though philanthropists promoted basic and creative research, most philanthropists seemed much distanced from ideas about their obligations to their grantees:

“They [the researchers] cannot demand anything. Yes, they cannot do anything to us.” Interviewee 71, Philanthropist

Or as another respondent put it:

“Actually they are accountable (…) To us.” Interviewee 20, Philanthropist

However, there were still some legal obligations of the philanthropists to the researchers, philanthropists would not act outside the law:

“We have actually embarked on a contractual relationship with them, so we’re committed to giving them our money if they say they’ll do what they do.” Interviewee 68, Philanthropist

The legal dimension also pertained to the tax authority, but such requirements also pinpointed the lack of obligations to the researchers beyond acting in a legally sound manner:

“We can give to exactly what we want, there is no one to question it other than the tax authority.” Interviewee 53, Philanthropist

The lack of obligations seemed to reside in all aspects except of the purely legal, and the legal dimension was the least present in the case of individual philanthropists. Here the entire relationship to the researchers was simultaneously detached and serendipitous:

“I write a check and I send it and then sometimes I go visit them.” Interviewee 4, Philanthropist

**Pattern summary:** Independently wealthy philanthropists were not commercially interested, and they rationalized their funding by speaking of the virtues of basic research, shying away from applied science. Among the philanthropists there was also a conspicuous lack of obligations to the researchers they funded, beyond respecting the limits of the law.

### NPF: Fundraising Dependent Nonprofits

**Contributions to society:** The fundraising dependent nonprofits told a story that differed from the philanthropists. Instead of focusing on basic science for its own sake, these funders viewed the patients as the primary population they served, rather than the researchers. This can, for example, be seen in this patient-focused nonprofit:

“We are a part of the chorus that’s saying that it’s important to fund this research for brain injured children and to try stem cell therapies in brain injured children.” Interviewee 10, Fundraising Dependent Nonprofit Funder

These fundraising dependent nonprofit funders’ view of research funding was usually highly applied, the funding should lead to tangible medical outcomes:

“I think it’s really important for everyone. I mean one in 25 people will die of a neuro-degenerative disease within the next 10 years, so it’s important that we use the best scientific tools that we can find of which stem cells are one to aggressively find cures for these diseases.” Interviewee 29, Fundraising Dependent Nonprofit Funder

However, in addition to telling the story of the patients, some fundraising dependent nonprofit funders also had a wish to show how that medical progress could help the economy at large:

“I think that having the corner on research in stem cells or aging is going to be very valuable for a location moving forward because their growth industries, I mean the importance of aging research is only going to continue to go up and up and up as more and more people get older. So having the expertise in the local area provides a boost to the economy.” Interviewee 5, Fundraising Dependent Nonprofit Funder

Some of these funders also explicitly emphasized the importance to move beyond basic research into the clinic:

“the research we are supporting is to be as close to the patient as possible. That means not to prioritize basic research in the first place, but that [research] which will benefit patients.” Interviewee 63, Fundraising Dependent Nonprofit Funder

Nonprofit disease focused funders pointed to the connections between the well-being of the patients and the cost benefits to society at large:

“thanks to stem cell research they will come up with effective treatments and then people will not have to retire early, but can work fully, which is good for society. Likewise, people who are older, who are no longer in the labor market, they will be able to remain in their own home and be independent and require less home care and help, which will also reduce the cost burden for society in terms of Parkinson’s.” Interviewee 66, Fundraising Dependent Nonprofit Funder

**Obligations to researchers:** In terms of the obligations of the fundraising dependent nonprofit funders to the researchers performing the research, they were rather similar to the philanthropists:

“persons who do not receive grants cannot exactly come and require correction (…) you cannot come and complain very much about the fund’s grant-making decisions.” Interviewee 55, Fundraising Dependent Nonprofit Funder

Like philanthropists, fundraising dependent nonprofits articulated a low degree of obligations, mainly based on the contractual relationship of the grant-giving:

“We’re accountable because of contractual relationships but that’s it.” Interviewee 3, Fundraising Dependent Nonprofit Funder

However, as the same respondent continued, there were also other dimensions of obligations that may matter to the fundraising dependent nonprofit funders. This related to the nonprofit’s aim to act consistently with the articulated purpose, standard and ethics of each specific organization:

“We’re obviously accountable on a basis of our own standards and ethics.” Interviewee 3, Fundraising Dependent Nonprofit Funder

This idea was echoed, albeit also delimited, by another fundraising dependent nonprofit funder:

“Some type of moral responsibility, they [the researchers] may require, but not otherwise.” Interviewee 74, Fundraising Dependent Nonprofit Funder

The moral responsibility of these fundraising dependent nonprofit funders to the researchers, mediated by their own organizational values, thus made them different from the philanthropists. But like the philanthropists, these organizations also asserted their independence to act according to their own whims and ideas in relation to the researchers they funded, and some even thought that this may make them a popular type of funder:

“Some of the criticism we sometimes get is that we may not be transparent in our assessments, we have no principle of public access (…). But it’s just so we have chosen [to do] it. (…) but on the other hand, I believe that we are popular.” Interviewee 45, Fundraising Dependent Nonprofit Funder

**Pattern summary:** Fundraising dependent nonprofits expressed slightly more obligations than independently wealthy philanthropists to the researchers they funded. They were also more geared towards applied research than philanthropists, keeping the patients in focus, which in the long run may also benefit society at large. Both types of NPF seemed to share little inclination to rationalize their contributions in commercial terms, but they had different views as to their respective focus on researchers and basic research (philanthropists) and patients (fundraising dependent nonprofits).

### Public Funders

**Contributions to society:** Differing very much from NPF, public funders were blatant in their usage of commercial arguments when describing their societal contributions:

“Well, we’re stimulating jobs [for] a start. We’ve been building buildings and we have been getting leverage funds from donors and so forth. So we have built, at one point, 1.1 or 1.2 billion dollars’ worth of buildings. (…) So, we are building jobs, so we are building buildings, building people coming into the space so they pay tax so this tax benefit is coming back to the state (…). So we have documented that and that is also on our website. Then, we will be making contributions which will be the biggest contributions we think will be benefits to the medical health so health savings.” Interviewee 13, Public Funder

The public funders did not only emphasize the commercial value of their funding, they also connected potential health benefits to the commercial advantages of the research:

“There are the sort of hard economic facts. So far we’ve created about 25,000 jobs. We are generating revenue in the form of income tax, sales tax through people who are in the stem cell business as a result of our funding. (…) [an] outside study commission that said we’d be generating about $200 million net (…) So there is the economic impact. Now, the real kicker will be if and when we hit on a therapy or a cure because at that point you’re now materially affecting state health issues, federal health, the spiraling costs that are getting worse and worse. And you can really have a huge economic impact if you come up with something that suddenly pulls a disease off the table, which we believe we will.” Interviewee 17, Public Funder

The commercial motivations were echoed also by other public funders:

“we hope that our efforts create small companies, seed companies of various types, who choose to pursue certain lines of development and thus multiply.”

Interviewee 72, Public Funder

The public funders described commercial success as a value in and of itself:

“We contribute to this through increased knowledge and innovation for types of hopefully new clinical therapies and also the possible commercialization that takes place in this. So innovation for us is not just a product, but an improved clinical management, and here we are convinced that the support for stem cell research will contribute to both.” Interviewee 78, Public Funder

**Obligations to researchers:** Looking at the relationship to the researchers funded, the public funders emphasized their obligations to their grantees:

“they [the researchers] can ask for accountability in terms of all processes being conducted in a correct manner.” Interviewee 77, Public Funder

These processes, for example, entailed:

“If we have kept our promises, if we have distributed our funding as we have said we would.” Interviewee 79, Public Funder

Also here it was the legal dimension that was most prominent:

“It’s a bit of a question of definition. They [the researchers] can do that [require the funder to have obligations to them] based on existing legislation, they cannot do it based on their own role.” Interviewee 78, Public Funder

Like the case with other funders, some researchers were upset by funding not granted. This was particularly attributed to the researchers’ scientific training:

“questioning they can always do, they’re scientists. (…) Yes, they make a hell of a noise and all that (…). Especially those who do not get money are trying to ask us to account for why they were not given when the others got [funding].” Interviewee 80, Public Funder

However, the interaction between the grantees and the public funders also could result in a highly interactive policy process, especially in cases when the grantees were research institutions rather than individual researchers:

“For every one of our grantees - our major grantees - I know the person or the institution or I know a lot of the people who are responsible for the oversight programs. I think what it creates is a dynamic where they are going to be more willing I think to engage in a policy process knowing that they can actually influence the policy. (…) if there is a rule that seems bureaucratic that they do not understand that is consuming resources but not accomplishing any social benefit, they can say you know I really do not understand this (…) so how can we change that. I think what that does is get them much more invested in the process knowing not only that they influence it but that they can influence it in a way that improves their operation.” Interviewee 16, Public Funder

**Pattern summary:** In comparison to NPF, public organizations had obligations to the researchers they funded. In terms of commercialization, the public funders did not shy away from rationalizations relating to commercial values and economic growth, instead these were very much emphasized. Both in terms of commercial rationalizations and obligations to researchers, public funders thus differed very much from NPF.

### Industry Funders

**Contributions to society:** Even though industry funders articulated their profit motives, many also chose to emphasize other sides of their activities. Quite counter-intuitively given their corporate form, they often began by speaking about health benefits and only then alluded to commercial gains. The order of the argument - first health then profit, was inverted to that of most of the public funders:

“Well, I think we are looking for a cure for a very important disease. The latest estimates, which I saw published in the Lancet in October, suggested that as of 2008, there were 347 million people in the world with diabetes and the rate was higher in the industrialized countries. To me, that says that what we are working could be a major benefit to the people (…) I think also just having the business here, you know, it’s a growing company, it creates jobs, it’s helping the investments as well.” Interviewee 32, Industry Funder

There were also instances when enthusiasm seemed to trump the drive for profit:

“I know everybody has to worry about the economics of keeping their companies going but I can - my sense is everybody who’s involved in these companies believe so passionately about what their therapies can mean for people but that’s really what keeps them going more than anything else. We went through a period of time a couple of years ago where you know it was very hard to raise money and the company was running on fumes and a number of people worked for free for months.” Interviewee 1, Industry Funder

Industry funders emphasized their contributions not only in terms of health gains, but also relating to standard setting and quality assurance:

“I think that we contribute to the quality assurance of this fairly tricky area, because we have gone through a baptism of fire during the years from ethical review boards, from public agencies, from our partners.” Interviewee 51, Industry Funder

Continuing on this more altruistic line of argument, some industry funders also aimed at helping society at large by creating educational opportunities:

“First of all, we provide education, some sort of training to do with embryonic stem cell research for free of charge, so any institute or university who wants to take on this research, we are willing to give out training and education for free and in a way I think that helps the country. Interviewee 38, Industry Funder

**Obligations to researchers:** Differing from the other funders, the industry funders were much more specific about their absolute and direct obligations to their grantees:

“[our] organization is accountable to its grantees. Very much.” Interviewee 22, Industry Funder

For industry funders, the legal and contractual dimension was not only relevant, it was key:

“Yes they [the researchers] can [demand things from the funder]. If you get the funding and have a contract, of course they can.” Interviewee 50, Industry Funder

Industry collaboration automatically entailed obligations to those granted funds:

“If we collaborate, then we (…) Then we are accountable.” Interviewee 47, Industry Funder

**Pattern summary:** Industry funders were inherently commercially geared, but also used other, not necessarily commercial rationalizations, in relating their contributions to society. In fact, industry funders used less commercial rationalizations than the public funders. Industry funders also emphasized their obligations to the researchers they funded. In this respect they were similar to public funders, and differed from NPF.

## Discussion

We have now delved into the interviews, seeing how research funders rationalized their funding in terms of their contributions to society and obligations to researchers. In this discussion section we will compare the results of our interviews and probe deeper into the patterns we have identified.

### NPF: Neither Rationalized nor Obliged

Our data reveals that the world society perspective seems to be primarily relevant to understand the development of industry, and even more public research funders, rather than NPF. The rhetoric of commercialization and economic growth barely permeates the rationalizations of the NPF, which indicates limits to the spread of world society. In addition, NPF’s lack of rationalizations related to commercialization and economic growth are not coupled to obligations to researchers, but quite the contrary. There is also a divide between the two groups of NPF, where fundraising dependent nonprofits, on the one hand, care about patients and potential health gains of the research whereas philanthropists, on the other hand, care about basic research and research for its own sake.

Philanthropists, out of all funders, are least interested in contributing to economic growth and commercialization. Here, the contributions instead entail improving medical research and medicine at large, but mainly in an abstract manner. It is among the philanthropists that we find the idea about science being important for its own sake (Merton 1973). Philanthropists also differ from the fundraising dependent nonprofits by not articulating a dedication to patients, and at the same time their dedication to the researchers seems very low, being restricted to legal requirements.

Even though philanthropists’ general lack of obligations to grantees differs from one case study of philanthropic research funding (Azoulay et al. 2011), it is very much in line with a broader body of work centered on the lack of mechanisms controlling the distribution of independent wealth. While these studies have not focused specifically on research funding, they also indicate little obligations to grantees. In this literature, independently wealthy philanthropists are claimed to differ from other funders, both public and private, as their own funding provision is not related to the evaluation of their philanthropic actions. They are therefore not obliged to account for their actions (Fleishman 2009; Frumkin 2006; Hess 2005; Weinryb 2015). This lack of obligations to grantees is by some disapproved and by others celebrated as a definitional characteristic of independently wealthy philanthropists (Ostrander 2007; Frumkin 2006; Prewitt 2006; Fleishman 2009). Independently wealthy philanthropists are free to choose how they spend their money, and as long as they do not infringe the law they are not dependent on anyone’s evaluation of their actions (Fleishman 2009; Prewitt 2006). Our findings seem to assert the independence of philanthropists, and also point to how their abstract ideas about funding basic and creative research are not necessarily anchored in any commitment to the researchers funded, beyond strictly contractual obligations. In addition, we see that not all philanthropists, especially not the individual donors, operate on a contractual basis.

Differing from philanthropists, fundraising dependent nonprofits have an applied slant to their framing of their societal contributions, but they are mainly committed to the patients, and also express rather few obligations to the researchers they fund. For these funders, while patients are put first, to some extent this dedication to the patients can in the long run contribute to society at large. Human embryonic stem cells may potentially treat and cure a number of enigmatic diseases, and it is those hopes and promises that lead the way for the fundraising dependent nonprofit funders. This is in line with the nonprofit literature, which discusses obligations to the clients served by the organization (Edwards and Hulme 1996; Ebrahim 2003; O’Dwyer and Unerman 2008). In our interviews we can note that the fundraising dependent nonprofits express obligations to their clients, in this case patients, and that researchers are less relevant to them. The obligations to a patient community may also be related to patients and their friends and families being the main target group in the nonprofits’ fundraising endeavors. The nonprofit literature also discusses these organizations’ obligations to themselves to act in line with their mission, goal, and values (Ebrahim 2003; Najam 1996). These types of obligations are also expressed in the interviews, where several respondents emphasize the importance of the nonprofits to act in a morally sound manner. The fundraising dependent nonprofits thus seem to carry some organizational ideals about who they are as organizations, and how they should act. Those ideals may also form their relationships to the researchers they fund, in this way creating some type of obligations to these grantees, albeit rather meager.

### Public and Industry Funders: Rationalized and Obliged

In contrast to NPF, public and industry funders are focused on commercialization and economic growth, while simultaneously expressing obligations to the researchers they fund. However, public rather than the industry funders are the most commercially geared when rationalizing their contributions to society. The world society literature (Meyer et al. 1997; Drori 2008) may offer an explanation to the somewhat counterintuitive results we see when comparing public and industry research funders. As funders are subjected to an ongoing process of globalization and rationalization, a diffusion of ideas where various sources amalgamate create rather uniform ideas about what an organization, of any kind, should look like, and how it should organize its activities (Bromley and Meyer 2014; Meyer and Bromley 2013). In this view, isomorphic rationalizations, such as commercialization and economic growth, are spreading throughout the world, streamlining seemingly diverse science organizations and rendering them legitimacy which will potentially facilitate their survival (Drori et al. 2006; Meyer and Rowan 1977). Given that public funders are not constituted as profit-driven, and therefore not as legitimate as such, they need to acquire the legitimacy provided by rationalizing their contributions as commercial. This need may not be as strong for the for-profit industry funders who instead wish to show that their profit gaining motives also contribute to the public sphere at large.

Yet, the commercial rhetoric of the public funders is interesting given that the main argument that has been used by proponents of human embryonic stem cell research has been related to saving lives through novel treatments rather than making profits (Hug 2006), especially in contexts where the ethical dimension of the research has been questioned. In instances when human embryonic stem cell research has been controversial on ethical grounds, it has been primarily justified by its potential benefits to patients. But instead of reverting to this type of health-based argument, many public funders are referring to immediate commercial gains and economic growth, such as the benefits generated by job creation. To understand their commercial framing of their contributions from an empirical perspective, it is important to keep in mind that the promised treatments that human embryonic stem cell research would deliver have not yet materialized (Bubela et al. 2012). The public research funders may thus prefer to phrase their contributions as immediate commercial advantages rather than health benefits, also because the health gains have not yet been realized, and the time frame of their realization is unclear. However, as some funders explain, long-term health benefits are also part of the plan, and these are also rationalized as generating future economic prosperity.

Our results show that it is actually in the industry-sponsored research, rather than among public funders, that the respondents articulate contributions beyond commercial rationalizations. For the industry funders, it seems *comme il faut* to demonstrate adherence to altruistic principles by referring to the greater good that the research is contributing to. This greater good is much more varied and nuanced than the contributions articulated by the NPF. Some previous research predicts that industry funders would prioritize commercial gains (Blumenthal et al. 1996; Glenna et al. 2011; Goldfarb 2008; Sismondo 2007). However, our interviews show that industry funders also refer to health gains, standard developments, and educational opportunities offered, and a general altruistic team spirit forsaking remuneration for the sake of the research. In this last case, the industry funders are narrating a story about industry-sponsored research where the researchers worked without salary for a certain period, based on their belief in the importance of their scientific work. This multitude of contributions can also be compared to more straight forward health gains articulated by the fundraising dependent nonprofits and the contribution to basic research being the main rationalization used by the philanthropists. Industry funders seem more tied to the research practice itself in comparison to the NPF, and this close connection provides them with a more complex array of rationalizations they can employ when articulating their contributions to society at large. This also points to the strong links between industry funding and academia, not necessarily only in the commercialization process itself, but also as two related by separate endeavors that converge among industry funders (Gulbrandsen and Smeby 2005; Murray 2002, 2004). The varied contributions stated by the industry funders also tell us something about the one-step-removed funding practice of some of the NPF, especially the philanthropists. Whereas many fundraising dependent nonprofits are up-to-date on the possible medical application of their particular disease-focused research, the philanthropists are vaguer and more removed not only from the researchers, but also from the research practice itself. This may also be somewhat related to the difference between sponsoring basic and translational research, directly aimed at the clinic.

An empirical explanation may also here be found in the start-up nature of the stem cell industry, where clinical applications are still to be realized (Bubela et al. 2012). In this early innovation stage, commercial funders need to articulate additional motivational cues beyond profit to harness the energy needed to get through the “valley of death,” where so many novel biomedical ventures have faced their end. The lack of clinical applications of human embryonic stem cell research may thus make the public funders even more commercially geared in aspects other than the immediate clinical results of the research itself, and may at the same time limit industry funders in their talk about commercial gains. The enthusiasm and non-commercial rationalizations of the industry funders can also be seen in light of these funders hoping to sell human embryonic stem cell treatments in a market where there is a strong constituency ethically opposing the research from a pro-life perspective. Here the rhetorical importance of the research beyond solely profit may be even more important.

## Conclusion

Using the framework provided by the world society literature of organizational institutionalism, we have analyzed the spread of rationalized notions of science funding based on a rhetoric of commercialization and economic growth. Our findings that public and industry funders are rather similar in their rationalizations, corroborates the world society notion of different types of organizations across the globe becoming more alike (Bromley and Meyer 2014; Meyer and Bromley 2013). However, although our study supports the basic premise that describes how rationalized ideas on how to organize travel freely across the globe, creating isomorphism in the way science and science funding is argued for (Meyer et al. 1997; Drori et al. 2003; Drori and Meyer 2006), our results also suggest that there are limits to this spread of rationalized concepts. Although the extreme commercialization of the public funders supports the world society literature, the lack of commercialization as a rationalization strategy among NPF also indicates limits to the spread of such concepts. This suggests that there may be “pockets” of research funders within this field that are somewhat less prone to rationalize this way, or perhaps less exposed to these pressures.

The lack of rationalizations related to commercial applications and economic growth among the NPF is somewhat surprising given research demonstrating that nonprofits, although not necessarily those funding research, are also increasingly commercially geared and employ the type of rationalized vocabulary that we are investigating here (Hwang and Powell 2009; Maier et al. 2016). We see three possible ways of understanding the extreme rationalization of public funders and the lack of such arguments among NPF.

Firstly, as discussed at the outset of this paper, NPF may differ from other research funders due to their form of resource provision (Weisbrod 1991; Hansmann 1980). This functional interpretation indicates that the lack of profit gaining motives of the NPF would make them less malleable to commercial pressures than other funders, and less in need of the legitimacy rendered by acknowledging the importance of commercialization and economic growth in their rationalizations. But although a functional argument can explain the lack of commercial rationalizations of the NPF, it does not tell us why public funders are the most rationalized in terms of the concepts examined here, despite them too being functionally different from industry. One way to understand the sharp difference between NPF and public funders is that new public management (Hood 1991), primarily aimed at public administration, may have made commercially tinted rationalizations more important for public funders than for NPF in their strive to create and retain legitimacy (Meyer and Rowan 1977).

A second way to understand these results is that the NPF appear to be rather decoupled from the field of science, expressing none or very limited obligations to the researchers that they fund and to the scientific pursuit as such. While public and industry funders recognize some obligations to the scientists – in terms of providing money, motivating their decisions, and caring about relations to grantees – NPF appear to perceive themselves as completely freed from such obligations. They are in this sense dis-embedded from the actors and activities that they fund, at least in comparison to other forms of funders, which may strengthen their ability to resist rationalization pressures. This is, however, a hypothesis that would need further empirical investigation to be verified. More specifically, more studies applying a comparative perspective like ours would help unpack further nuances in the approach and perspective of different forms of funders, including NPF. Unlike the main body of comparative work conducted this far, focusing on large cross-societal comparisons of philanthropic foundations and nonprofit organizations in different countries (Anheier 2001; Salamon and Anheier 1997; Anheier and Daly 2007), we suggest that detailed comparisons of NPF to other types of research funders would enhance the understanding of the spread, or not, of rationalized concepts in the field of science and science funding. For instance, as suggested earlier, investigating the extent to which NPF follow the rationalized principles of accountability – or holding their grantees responsible for results and how they spend the money – would significantly add to our understanding of the ability of NPF to resist or decouple from rationalized notions of science funding. This would add nuances to the understanding of the position of NPF as “free” or independent actors in this field.

A third explanation may be that the results we see in our study are idiosyncratic to the extreme case selection. Human embryonic stem cell research funding is a suitable empirical topic for our study as it is a high-profile field where all funders are likely to have had conscious motives for their funding practices. However, the many controversies in the field influenced research funding and research collaborations (Furman et al. 2012; Scott et al. 2010, 2011), and these dynamics aggravate the possibility of interpreting our results. The controversial nature of the research, which prompted the involvement of NPF, might also have influenced the usage of commercially tinted rationalizations among all funders, albeit in different ways. This caveat is important in relation to the generalizability of our results. Even though possible idiosyncrasy should be taken into account in all research, especially qualitative work, the extreme case selection makes it even more important. The benefit of our extreme case is that it reveals interesting differences, but future research will have to test and validate the generalizability of these results on a larger scale.

The results presented here suggest that more research is needed in order to better understand the distinctive role and position of NPF in the field of science funding, and the implications the spread of rationalizations, and lack thereof, may have on the field of science. One apparent way forward is to focus on other concepts or notions that follow ideals of rationalized science funding actors, and to what extend NPF differ in relation to these. Furthermore, we may ask why this variation between types of funders matters? Although the descriptive nature of our study points to differences in rationalizations between types of research funders, it does not provide any information about the effects these rationalizations may have. We have indicated that avoidance of commercial ideals among NPF appears to come at the price of less obligations to researchers, which may potentially have policy implications. However, the relationship between research funders guarding the integrity of science and the spread of rationalized commercially tinted ideals is unclear and our knowledge about it is inconclusive. We hope that future studies can help to explore the implications of our findings in terms of the consequences for researchers and research practices.
